# Novel Heating-Induced Reversion during Crystallization of Al-based Glassy Alloys

**DOI:** 10.1038/srep46113

**Published:** 2017-04-13

**Authors:** F. F. Han, A. Inoue, Y. Han, F. L. Kong, S. L. Zhu, E. Shalaan, F. Al-Marzouki, A. L. Greer

**Affiliations:** 1 School of Materials Science and Engineering, Tianjin University, Tianjin, 300072, China; 2 International Institute of Green Materials, Josai International University, Togane, 283-8555, Japan; 3 Department of Physics, King Abdulaziz University, Jeddah, 22254, Saudi Arabia; 4 MISiS, National University of Science and Technology, Moscow, 119049, Russia; 5 Department of Materials Science and Metallurgy, University of Cambridge, Cambridge CB3 0FS, UK

## Abstract

Thermal stability and crystallization of three multicomponent glassy alloys, Al_86_Y_7_Ni_5_Co_1_Fe_0.5_Pd_0.5_, Al_85_Y_8_Ni_5_Co_1_Fe_0.5_Pd_0.5_ and Al_84_Y_9_Ni_4_Co_1.5_Fe_0.5_Pd_1_, were examined to assess the ability to form the mixture of amorphous (am) and fcc-aluminum (α-Al) phases. On heating, the glass transition into the supercooled liquid is shown by the 85Al and 84Al glasses. The crystallization sequences are [am] → [am + α-Al] → [α-Al + compounds] for the 86Al and 85Al alloys, and [am] → [am + α-Al + cubic Al_*x*_M_*y*_ (M = Y, Ni, Co, Fe, Pd)] → [am + α-Al] → [α-Al + Al_3_Y + Al_9_(Co, Ni)_2_ + unknown phase] for the 84Al alloy. The glass transition appears even for the 85Al alloy where the primary phase is α-Al. The heating-induced reversion from [am + α-Al + multicomponent Al_*x*_M_*y*_] to [am + α-Al] for the 84Al alloy is abnormal, not previously observed in crystallization of glassy alloys, and seems to originate from instability of the metastable Al_*x*_M_*y*_ compound, in which significant inhomogeneous strain is caused by the mixture of solute elements. This novel reversion phenomenon is encouraging for obtaining the [am + α-Al] mixture over a wide range of high temperature effective for the formation of Al-based high-strength nanostructured bulk alloys by warm working.

Considering current priorities for energy saving, it is important to develop high-strength and low-density materials with high values of elevated-temperature strength, ductility, corrosion resistance, wear resistance and oxidation resistance. Nanocomposite (fcc-Al + compounds) bulk alloys of Al-Mm-Ni-Zr (Mm = mischmetal), produced by warm extrusion of amorphous and fcc-aluminum [am + α-Al] phase mixtures, exhibit high tensile yield strengths of 600–800 MPa and fracture strengths of 800–1000 MPa, together with plastic elongation of 2–5%^1^,^2^,^3^, and high elevated-temperature strengths of 210 MPa at 573 K and 100 MPa at 673 K^4^. The successful development of such high-strength Al-based bulk alloys is attributed to the utilization of [am + α-Al] phase mixtures resulting from rapid solidification. It is important to clarify whether the [am + α-Al] phase mixture can be maintained over a wide range of extrusion temperature as well as at high solute contents of about 15 at%. The precipitation of compounds occurs easily at high temperature and at high solute content, and hinders the formation of fully consolidated bulk alloys by secondary working such as warm extrusion. The [am + α-Al] phase mixture is formed for Al contents above 86 at.% Al^5^, and the slight decrease in Al content to 85 at.% causes precipitation of a [α-Al + compounds] phase mixture during primary crystallization of the glass^6^. For Al contents below 85 at%, there are no reports of the formation of [am + α-Al] phase mixtures at temperatures higher than primary crystallization. The formation of [am + α-Al] phase mixtures at the high temperatures associated with warm working could be expected to enable the processing of a new type of Al-based nanostructured bulk alloys with useful characteristics.

The formation of [am + α-Al] phase mixtures has been studied under a wide range of casting and annealing conditions because of its engineering importance^7^. In the present study of Al-Y-Ni-Co-Fe-Pd glasses, we find a heating-induced reversion in the 84Al glass. In this reversion, the multicomponent cubic compound Al_*x*_M_*y*_ (M = Y, Ni, Co, Fe, Pd) disappears and the volume fraction of residual amorphous phase increases. This unusual, apparently inverted, transformation leads to the retention of a [am + α-Al] phase mixture over a wide temperature range up to 703 K. Given the potential importance of this finding for the future development of engineering Al-based nanostructured bulk alloys with high elevated-temperature strength, in the present work we aim to elucidate the mechanism of this transformation, and to identify the conditions under which it can be achieved.

## Results

### Annealing-induced structural change

**Figure 1:**
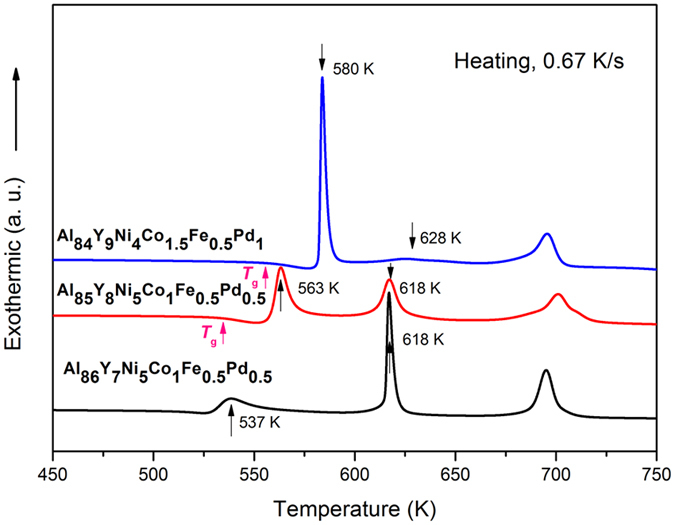
Differential scanning calorimetry curves of the melt-spun 86Al, 85Al and 84Al amorphous alloys. The glass-transition temperatures *T*_g_ and peak temperatures of the exotherms are marked.

Figure 1 shows DSC curves obtained on heating the 86Al, 85Al and 84Al glasses. The curve for the 86Al alloy has a very broad exothermic peak showing the first stage of crystallization, followed by two further sharp exothermic peaks. In contrast, the 85Al and 84Al alloys first show the glass transition on heating, followed by a narrow supercooled liquid region and then three exothermic peaks. The temperatures of the second exothermic peak are similar for the 85Al and 86Al glasses, and the temperatures of the third exothermic peak are similar for all three glasses, indicating some similarity of the heating-induced crystalline phases. However, the first and the second exothermic peak temperatures for the 84Al glass are different from those for the other glasses. Overall, the glass-transition temperature *T*_g_ and the temperature of the first crystallization peak *T*_x_ increase with decreasing Al content.

**Figure 2:**
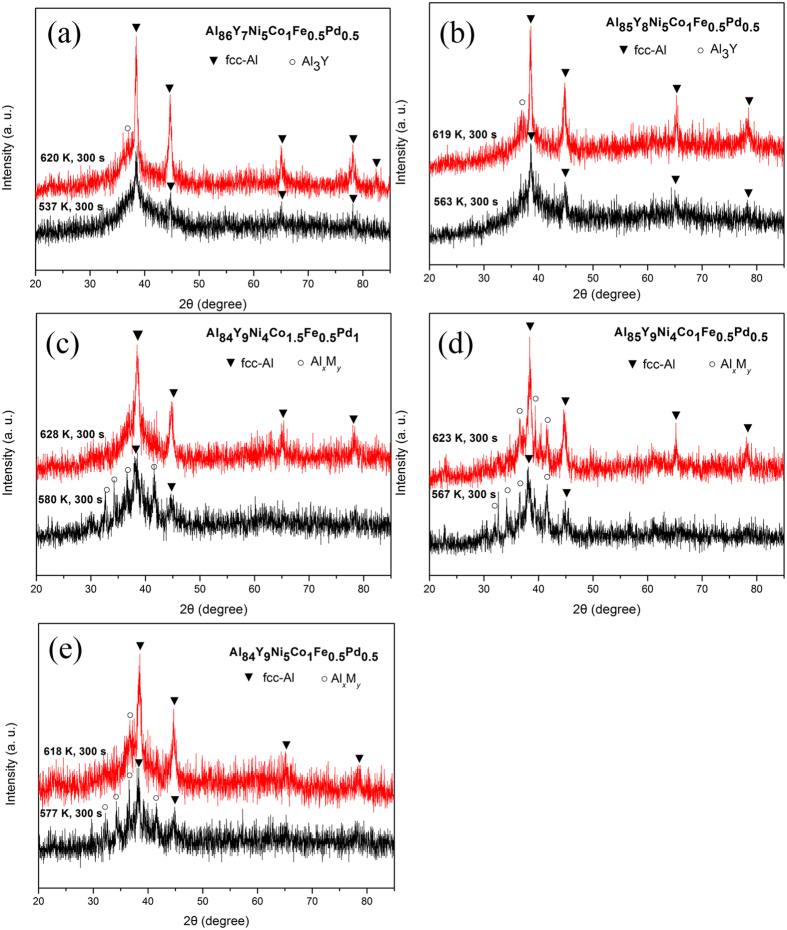
X–ray diffraction patterns of the (**a**) 86Al, (**b**) 85Al and (**c**) 84Al glasses annealed for 300 s at each peak temperature of the first and the second exothermic peaks shown in Fig. 1, (**d**) Al_85_Y_9_Ni_4_Co_1_Fe_0.5_Pd_0.5_ and (**e**) Al_84_Y_9_Ni_5_Co_1_Fe_0.5_Pd_0.5_ glasses at each peak temperature of their first and second exothermic peaks.

Samples of each glass were held for 300 s at the peak temperatures of the first and second exotherms, and X-ray diffraction was used to identify the phases associated with each peak (Fig. 2(a) and (c)). Primary crystallization of the 86Al and 85Al glasses gives α-Al, and the overall sequence [am] → [am + α-Al] → [α-Al + compounds] is consistent with previous data for Al-based glasses^5^,^8^. In contrast, crystallization of the 84Al glass proceeds through [am] → [am + α-Al + Al_*x*_M_*y*_] for the first peak and [am + α-Al + Al_*x*_M_*y*_] → [am + α-Al] for the second peak. The first stage can be regarded as a eutectic reaction. This reaction is not completed in the first exotherm, presumably because the element redistribution from Al to Al_*x*_M_*y*_ is suppressed by the very low mobility of Y and Pd which have large negative heats of mixing with Al. In the second exotherm of the 84Al glass, the multicomponent Al_*x*_M_*y*_ compound disappears and there is a distinct increase in the volume fraction of amorphous phase. As shown in Fig. 2(d), a similar reversion is found on heating Al_85_Y_9_Ni_4_Co_1_Fe_0.5_Pd_0.5_ and Al_84_Y_9_Ni_5_Co_1_Fe_0.5_Pd_0.5_ glasses. Besides, the DSC curve of the latter alloy is shown in supplementary Fig. 1. The similar heating-induced reversion phenomenon was recognized from the DSC data. This type of phase transformation is unexpected, and seems not to have been reported before in amorphous alloys.

**Figure 3:**
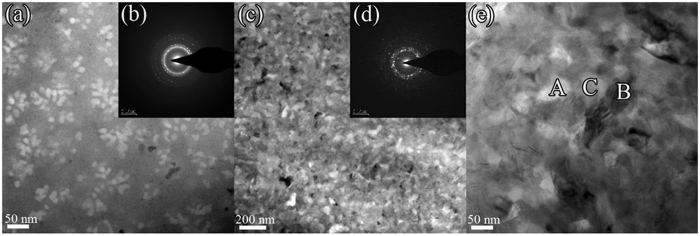
(**a**) Bright-field TEM image, (**b**) selected-area electron diffraction pattern of the 85Al alloy annealed for 300 s at a temperature just above the first exothermic peak, and (**c**) bright-field TEM image, selected-area electron diffraction pattern (**d**) and enlarged bright-field TEM image (**e**), of the Al_84_Y_9_Ni_4_Co_1.5_Fe_0.5_Pd_1_ initially fully glassy alloy annealed for 300 s at 580 K corresponding to the first exothermic peak. Areas A, B and C are identified as α-Al, Al_*x*_M_*y*_ compound and residual amorphous phase respectively.

Figure 3 shows (a) a bright-field TEM image and (b) a selected-area electron diffraction pattern for the 85Al glass annealed for 300 s at a temperature just above the first exothermic peak. The primary α-Al crystals are globular-dendritic with a size of 30–60 nm. In contrast, in the 86Al glass, the primary α-Fe crystals are spheroidal and much smaller (~15 nm). The morphology and size of the primary crystals appear to be strongly influenced by the type of matrix phase in which they form, a supercooled liquid for 85Al or a solid glass for 86Al. The larger atomic mobility in the supercooled liquid naturally leads to a coarser microstructure.

**Figure 4:**
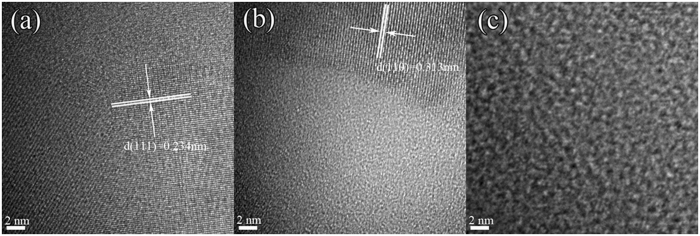
High resolution HREM images (**a**), (**b**) and (**c**) taken from regions A, B and C, respectively, in the enlarged bright-field TEM image shown in Fig. 3(e).

A relatively coarse primary microstructure formed in the supercooled liquid is also found for the 84Al alloy. Figure 3 shows (c) a bright-field TEM image, (d) a selected-area electron diffraction pattern, and (e) an enlarged TEM image for a sample annealed for 300 s at 580 K, corresponding to the peak temperature of the first exotherm. The microstructure has α-Al, cubic Al_*x*_M_*y*_ and some residual amorphous phase, marked A, B and C, respectively, in Fig. 3(e). HREM images (Fig. 4) from each type of region show contrast and lattice spacings confirming that A, B and C are α-Al, cubic Al_*x*_M_*y*_ and amorphous phase, respectively. From EDX analysis, the average composition of the grains taken to be Al_*x*_M_*y*_ is approximately Al_71_Y_14_Ni_6.7_Co_2.8_Fe_1.3_Pd_4.2_, indicating that the compound is enriched in solute M elements, particularly Y.

**Figure 5:**
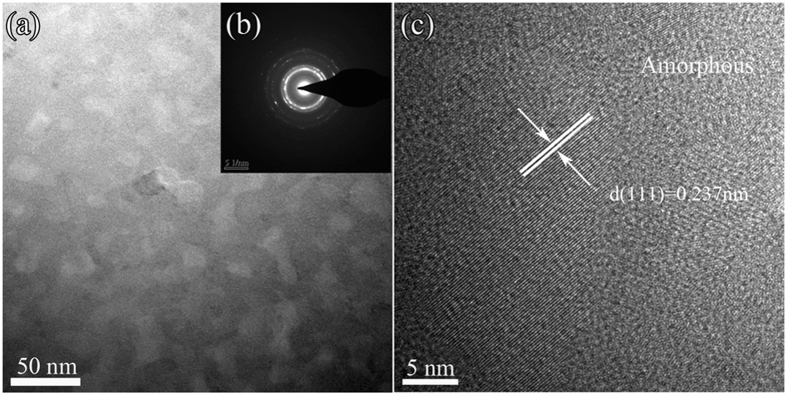
Bright-field TEM image (**a**), selected-area electron diffraction pattern (**b**) and HREM image (**c**) of the initially fully glassy alloy Al_84_Y_9_Ni_4_Co_1.5_Fe_0.5_Pd_1_ annealed for 300 s at 620 K corresponding to the second exothermic peak (Fig. 1).

Figure 5 shows (a) a bright-field TEM image, (b) a selected-area electron diffraction pattern, and (c) a HREM image of the 84Al alloy annealed for 300 s at 628 K corresponding to the peak temperature of the second exotherm. Bright-field TEM (Fig. 5a) shows a microstructure that appears to consist of only two phases, and is much simpler than that in the same glass annealed at 580 K. The diffraction pattern consists of sharp rings and broad haloes, indicating α-Al and amorphous phases. HREM (Fig. 5c) shows regions with lattice-fringe contrast (α-Al) and with irregular modulated contrast (amorphous phase). Based on the results in Figs 3 and 5, the first exotherm is attributed to the transformation [am] → [am + α-Al + Al_*x*_M_*y*_], and the second exotherm to [am + α-Al + Al_*x*_M_*y*_] → [am + α-Al], in agreement with the X-ray diffraction identification of the phases (Fig. 2c).

The lattice parameter of the α-Al phase generated in the first and second exotherms was evaluated from the X-ray and electron diffraction patterns. In the 84Al alloy the parameter was measured to be 0.405 nm after the first transformation and 0.406 nm after the second, being slightly larger, especially in the latter case, than the parameter (0.404 nm) for pure Al^9^. This suggests that the α-Al formed by heating-induced reversion contains more solute elements, especially Y, compared with that formed in the first exotherm. Considering the sequence of atomic sizes Y > Al > Pd > Co,Ni > Fe^10^, it is likely that Y plays the dominant role in increasing the lattice parameter, even when other solutes are present in the α-Fe.

### Annealing-induced hardness change

**Figure 6:**
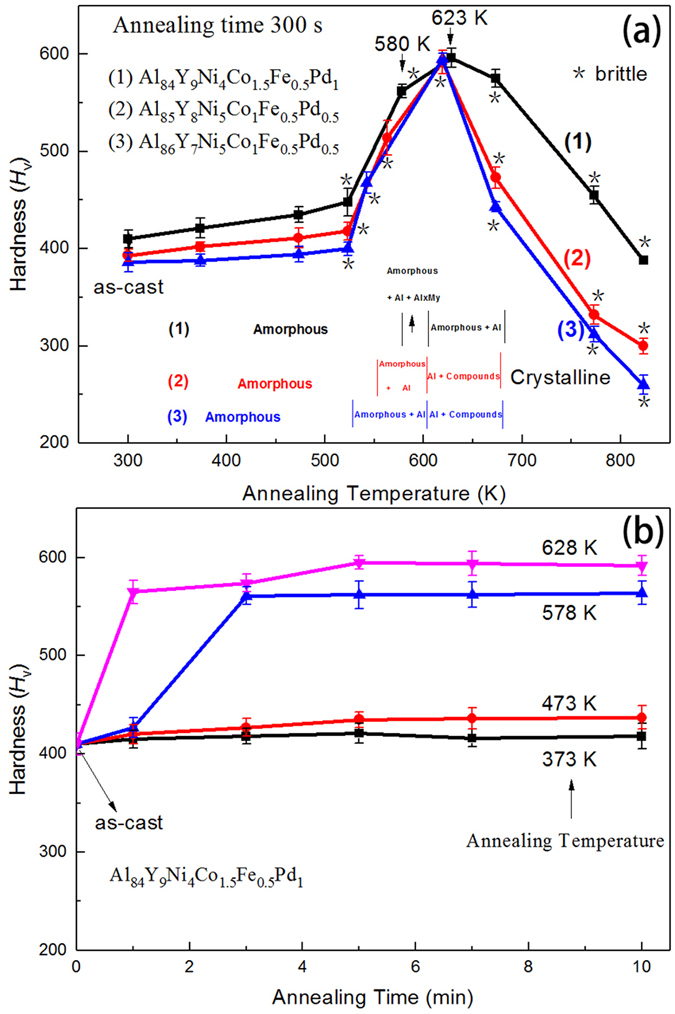
Vickers hardness as a function of (**a**) isochronal annealing temperature for the initially fully glassy 86Al, 85Al and 84Al alloys and (**b**) time of annealing at the indicated temperatures of the initially fully glassy 84Al alloy.

Figure 6(a) shows the change in Vickers hardness (*H*_V_) with annealing temperature for the three Al-based glasses. The *H*_V_ value in as-quenched state increases in the order 86Al < 85Al < 84Al. The *H*_V_ values of the 86Al and 85Al alloys increase very slightly for annealing up to 525 K, increase rapidly with further increase in annealing temperature, show a maximum of ~593 for annealing at 619 K, corresponding to the second exotherm, and then decrease significantly for higher-temperature anneals. The *H*_V_ of the 84Al alloy, in contrast, increases somewhat more in the amorphous single-phase range, then increases rapidly for anneals at temperatures corresponding to the first exotherm, and reaches a maximum of 595 on annealing at the temperature of the second exotherm.

To investigate the possibility of obtaining higher hardness for the present Al-based alloys, the evolution of Vickers hardness with time at different annealing temperatures was measured (Fig. 6(b)). Independent of annealing temperature, the hardness values increase rapidly at first and then saturate. The saturated value is strongly dependent on annealing temperature. These changes in hardness agree with behavior previously reported for Al_88_Ni_4_Y_8_ glass^11^. The time to reach the saturated hardness value decreases with increasing annealing temperature. The maximum *H*_V_ values are nearly the same for the three alloys, although the constituent phases are [α-Al + compounds] for 86Al and 85Al, but [α-Al + amorphous phase] for 84Al. When the [am + α-Al] phase mixture decomposes in the third exotherm to [α-Al + Al_3_Y + Al_9_ (Co, Ni)_2 + _unknown phase], there is a significant decrease in *H*_V_.

## Discussion

The DSC curves (Fig. 1) do not show any glass transition for the 86Al alloy. The glass transition and a supercooled liquid region before crystallization are seen for both the 85Al and 84Al alloys, despite the difference in primary crystallization, to [am + α-Al] for 85Al, and to [am + α-Al + Al_*x*_M_*y*_] for 84Al. The appearance of the glass transition thus seems independent of the primary crystalline phases, though dependent on the thermal stability of the amorphous phase, which is dominated by the extent and nature of the solute additions to the aluminum. The relationship between the appearance of glass transition and the nature of primary crystallization is, however, likely to be important in understanding the formation and stability of Al-based glasses.

As already noted, the crystallization sequence of the 86Al and 85Al glasses in the present work is consistent with many previous observations^8^. The sequence in the 84Al glass is, however, entirely novel. The apparently back-to-front transformation from [am + α-Al + Al_*x*_M_*y*_] to [am + α-Al] can be regarded as a heating-induced peritectic decomposition of the Al_*x*_M_*y*_ compound, accompanied by the reversion to a higher volume fraction of amorphous phase and the growth of α-Al with a higher solute content than that formed in the first stage of crystallization. This transformation in which the volume fraction of amorphous phase increases is also exothermic; the transformation is therefore not directly analogous to melting. It is notable that the heat of reaction is so small.

This reversion transformation is significantly dependent on Y content. When the Y content is increased from 8 to 9 at.% in glasses with the same Al content of 85 at.%, a similar reversion phenomenon is seen (Fig. 2(d)). Besides, for the present alloys with compositions of Al_84–86_Y_7–9_Ni_4–5_Co_1–1.5_Fe_0.5_Pd_0.5–1_, the heating-induced reversion phenomenon was always observed only for the 9%Y-containing alloys and is not dominated by the change in Ni, Co and Pd contents, though the composition ranges of the three elements are limited. Thus, the reversion transformation may be concluded to require the dissolution of high Y content above 9%Y. Considering that Y has the largest atomic size of all the constituent elements and that the Y-Al and Y-TM atomic pairs have large negative heats of mixing^12^, an increase in Y content is expected to cause an increase in inhomogeneous strain in the Al_*x*_M_*y*_ compound, leading to it having a higher stored energy and increased instability. Thus, instability of the compound, and reversion, may be favored in alloys that: (1) are multicomponent, with (2) significant atomic size mismatches, and (3) pairs of atomic species with large negative heats of mixing, and (4) show a particular type of primary crystallization from the supercooled liquid. The first three of these criteria are similar to the three rules for the formation of bulk glassy alloys^13^,^14^,^15^. The high level of multiple solutes in the Al_*x*_M_*y*_ compound (measured by EDX, as noted earlier) may also give high-entropy effects favorable for amorphization^16^. The precipitation of such a multicomponent compound needs atomic ordering of the constituent elements; this ordering, difficult in the solid glass, can occur only in crystallization from the supercooled liquid. The presence of a fast-diffusing element is known to be important for the formation of amorphous alloys through solid-state reactions^17^,^18^. In the present alloys, iron has the smallest atomic size as well as much smaller negative heats of mixing (−1 to −2 kJ/mol) with the other TM elements as compared with the other atomic pairs^12^. Thus fast diffusion of Fe atoms may also assist the heating-induced amorphization reaction.

If the Al_*x*_M_*y*_ compound is so unstable as to undergo reversion, it is important to consider why it forms in primary crystallization. This may be associated with the difficulty of redistributing the solute elements in the low-temperature range. The ultimate reversion may aided by complex, multicomponent effects, raising the energy of the Al_*x*_M_*y*_ compound through internal inhomogeneous strains, and lowering the relative free energy of the amorphous phase.

**Figure 7:**
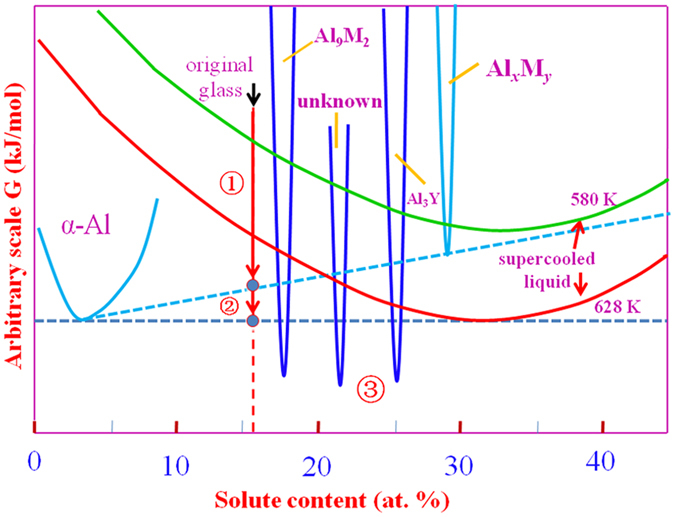
The relative free energies of the phases relevant for the crystallization of Al_84_Y_9_Ni_4_Co_1.5_Fe_0.5_Pd_1_ glass. In this schematic figure, the alloy is treated as a binary (Al + solute) system. The primary (arrow 1) and secondary (arrow 2) crystallization reactions are shown; ultimately (point 3) the stable phases are attained on heating.

Figure 7 shows the relative free energies as a function of composition for the various phases relevant for the first and second exotherms on heating the 84Al alloy. In this schematic picture, the glass is a binary system, with all solutes considered together. The free energy of all the phases decreases with increasing temperature, but that of the supercooled liquid decreases faster because of its higher entropy. In Fig. 7, the free energies of the crystalline phases are shown fixed, and relative to them the free energy of the supercooled liquid is shown for the temperatures of the peaks of the first and second exotherms. In the first exotherm, the free energy is lowered (arrow 1 in the figure) by eutectic crystallization to α-Al and Al_*x*_M_*y*_. In the second exotherm, the free energy is further lowered (arrow 2 in the figure) by the peritectic decomposition of Al_*x*_M_*y*_ to α-Al and supercooled liquid. On further heating there would be sufficient atomic mobility to permit formation of the equilibrium phases (marked 3 in the figure): Al_9_(Co,Ni)_2_, Al_3_Y and an unknown phase.

As noted earlier, lattice parameter measurements suggest that the α-Al formed in the second exotherm has a higher solute content than that formed in the first exotherm. This is opposite to the effect shown for α-Al in Fig. 7, and shows the limitations of treating the 84Al alloy as a binary system. In particular, a distinction can be made between the slow (Y, Pd) and the fast (Ni, Co, Fe) diffusing species. In the first exotherm, the Y and Pd may remain largely in the residual amorphous phase.

The formation of an amorphous phase through solid-state reaction has been reported in various alloy systems, e.g., in mechanically alloyed or sputtered bcc-CrTi^19^,^20^, and in sputter-deposited metastable Au_4_Ti + AuTi_3,_ Cr_2_Ti + α-Ti and Au_2_Ti + AuTi thin films^21^,^22^,^23^,^24^. The amorphization mechanisms in these cases may involve ‘inverse melting’ in which the amorphous phase has a lower entropy than its crystalline counterpart; they are therefore different from that in the present work, which is in some ways similar to partial peritectic melting on heating.

**Figure 8:**
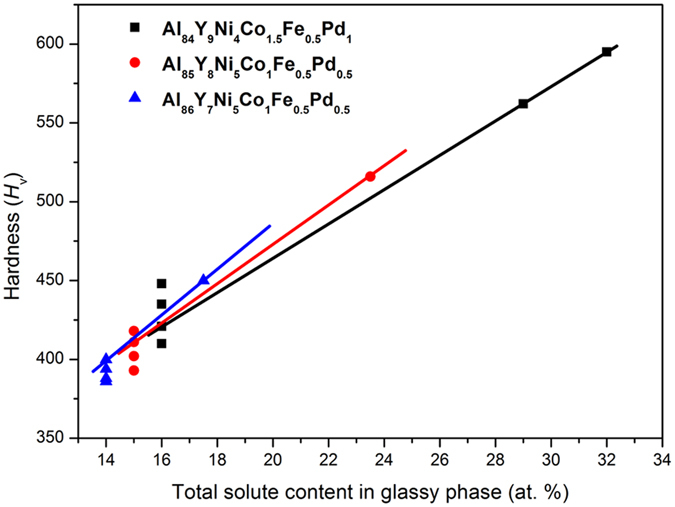
Vickers hardness of fully and partially amorphous Al-based alloys as a function of the estimated solute content in the amorphous phase after various annealing treatments.

The hardness *H*_V_ is highest (Fig. 6(a)) for [α-Al plus residual amorphous phase] mixtures obtained by annealing for 300 s at a temperature just above *T*_x2_. The maximum *H*_V_ value of ~595 is ~40% higher than those of the as-spun glasses. In earlier studies of Al-based glasses and partially crystalline nanocomposites formed from them, the Vickers hardness shows a good linear relation with the total solute content in the amorphous phase^11^,^25^. For the alloys in the present work, the total solute content in the amorphous phase in different annealed states was examined by EDX analyses. Figure 8 suggests that, across all the alloys in the present study, the hardness depends linearly on the solute content of the amorphous phase. In the 84Al alloy, the peak hardness can then be seen to arise by the solute enrichment resulting from the special mechanism of heating-induced reversion of the Al_*x*_M_*y*_ compound.

The maximum hardness seen in Fig. 6(a) shows that with novel nanoscale phase mixtures, Al-based alloys can have strengths much higher than obtainable conventionally. A key factor in realizing bulk alloys with optimum microstructures is retention of the binary phase mixture [amorphous + α-Al] during warm extrusion. If the extruded bulk alloys retain the binary phase mixture, and have an elastic strain limit above 0.2%, their yield strength is expected^26^ to be as high as ~(*H*_V_/3) × 9.8 ≃ 2000 MPa; this would constitute a new class of lightweight engineering material with exceptionally high values of yield strength, hardness, stiffness, wear resistance and elevated-temperature strength.

## Conclusions

On continuous heating, the crystallization of three glassy alloys, Al_84_Y_9_Ni_4_Co_1.5_Fe_0.5_Pd_1_, Al_85_Y_8_Ni_5_Co_1_Fe_0.5_Pd_0.5_ and Al_86_Y_7_Ni_5_Co_1_Fe_0.5_Pd_0.5_ (at.%), shows sequences of phase formation: [am] → [am + α-Al] → [α-Al + Al_3_Y + Al_3_Ni] for the 86Al and 85Al alloys, and [am] → [am + α-Al + Al_*x*_M_*y*_] → [am + α-Al] → [α-Al + Al_3_Y + Al_9_ (Co, Ni)_2_ + unknown phase] for the 84Al alloy. The primary [α-Al + Al_*x*_M_*y*_] eutectic-like crystallization for the 84Al alloy does not proceed to completion because of the difficult redistribution of solute elements, resulting in residual amorphous phase. The glass transition into the supercooled liquid was observed for the 85Al and 84Al alloys and hence there is no close relation between the appearance of the glass transition and the phases formed in primary crystallization. The heating-induced reversion from a [am + α-Al + cubic Al_*x*_M_*y*_] phase mixture to [am + α-Al] in the 84Al alloy is the first evidence for such a phenomenon in Al-based glasses. The similar reversion transformation is also observed for other Al_85_Y_9_Ni_4_Co_1_Fe_0.5_Pd_0.5_ and Al_84_Y_9_Ni_5_Co_1_Fe_0.5_Pd_0.5_ glasses containing 9%Y and hence the novel phenomenon is concluded to be mainly dominated by Y concentration. This instability of the multicomponent Al_*x*_M_*y*_ compound is likely to be associated with internal inhomogeneous strain due to the mismatch of solute-atom sizes. Entropic effects from the multicomponent composition may also promote this reversion. The formation of the metastable multicomponent Al_*x*_M_*y*_ compound is likely due to the difficulty of achieving the atomic rearrangements required for the formation of more stable compounds. The formation of the [am + α-Al] phase mixture by the heating-induced reversion of the unstable Al_*x*_M_*y*_ compound over a wide range of high temperature is promising in that it provides an opportunity to develop a new class of high-strength Al-based alloy.

## Methods

The compositions Al_86_Y_7_Ni_5_Co_1_Fe_0.5_Pd_0.5_, Al_85_Y_8_Ni_5_Co_1_Fe_0.5_Pd_0.5_ and Al_84_Y_9_Ni_4_Co_1.5_Fe_0.5_Pd_1_ (at.%) were chosen because, in glasses of this alloy system, the primary crystallization product changes from single-phase α-Al to a [α-Al + compounds] phase mixture at around 85 at.% Al^5^. Other two alloys containing 9%Y, Al_85_Y_9_Ni_4_Co_1_Fe_0.5_Pd_0.5_ and Al_84_Y_9_Ni_5_Co_1_Fe_0.5_Pd_0.5_ were also used for comparison. Alloy ingots were prepared by arc-melting a mixture of pure metallic elements with purities above 99 wt.% under an argon atmosphere. Melt-spinning of alloy liquid melted in a fused-silica crucible by high-frequency induction heating gave glassy ribbons with a thickness of about 25 μm and a width of about 1.5 mm. The as-quenched structures were examined by X-ray diffraction and transmission electron microscopy (TEM). The effects of heating were studied using differential scanning calorimetry (DSC, Perkin Elmer PM8000 with heating rate of 0.67 K/s), X-ray diffraction (Cu Kα), TEM with linked energy dispersive X-ray (EDX) spectroscopy, and high resolution electron microscopy (HREM). Hardness was measured using a Vickers hardness indenter with a load of 0.245 N.

## Additional Information

**How to cite this article**: Han, F. F. *et al*. Novel Heating-Induced Reversion during Crystallization of Al-based Glassy Alloys. *Sci. Rep.* **7**, 46113; doi: 10.1038/srep46113 (2017).

**Publisher's note:** Springer Nature remains neutral with regard to jurisdictional claims in published maps and institutional affiliations.

## Supplementary Material

Supplementary Information
