# Supplementary material for: Novel Heating-Induced Reversion during Crystallization of Al-based Glassy Alloys
Source: Sci Rep. 2017 Apr 13;7:46113. doi: 10.1038/srep46113 (PMC5390259; doi:10.1038/srep46113)
Supplement: Supplementary Information [file srep46113-s1.doc]

**Novel Heating-Induced Reversion during Crystallization of Al-based Glassy Alloys**

F. F. Han^1^, A. Inoue^1-4 *^, Y. Han^1^, F.L. Kong^2^, S.L. Zhu^1^, E. Shalaan^3^, F. Al-Marzouki^3^, A.L. Greer^5^

^1^School of Materials Science and Engineering, Tianjin University, Tianjin, 300072, China

^2^International Institute of Green Materials, Josai International University, Togane, 283-8555, Japan

^3^Department of Physics, King Abdulaziz University, Jeddah, 22254, Saudi Arabia

^4^MISiS, National University of Science and Technology, Moscow, 119049, Russia

^5^Department of Materials Science and Metallurgy, University of Cambridge, Cambridge CB3 0FS, UK

* corresponding author: [inoue@jiu.ac.jp](mailto:inoue@jiu.ac.jp)

Tel: +81-475-53-2166

Fax: +81-475-55-8811


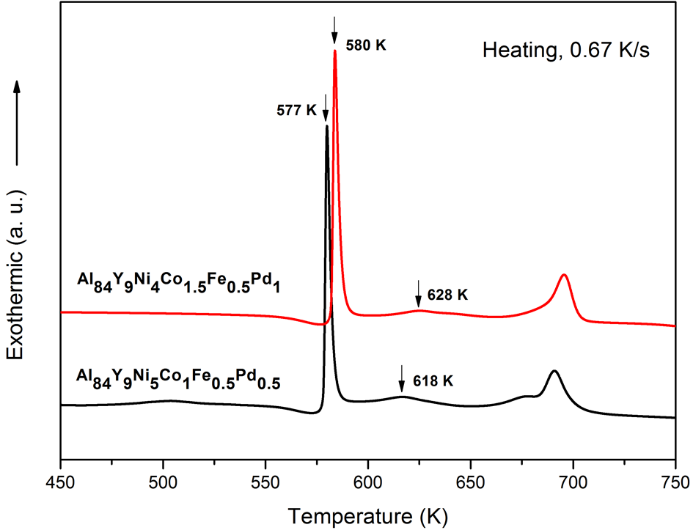


Fig. 1 DSC traces of melt-spun Al_84_Y_9_Ni_5_Co_1_Fe_0.5_Pd_0.5_ and Al_84_Y_9_Ni_4_Co_1.5_Fe_0.5_Pd_1_alloy ribbons.
